# A Systematic Review of Resilience Factors for Psychosocial Outcomes During the Transition to Adulthood Following Childhood Victimisation

**DOI:** 10.1177/15248380211048452

**Published:** 2021-10-20

**Authors:** Rachel M. Latham, Joanne B. Newbury, Helen L. Fisher

**Affiliations:** 1 Social, Genetic & Developmental Psychiatry Centre, Institute of Psychiatry, Psychology & Neuroscience, 4616 King’s College London, London, UK; 2 ESRC Centre for Society and Mental Health, 4616 King’s College London, London, UK; 3 Bristol Medical School: Population and Health Sciences, 1980 University of Bristol, Bristol, UK

**Keywords:** bullying, emerging adulthood, psychosocial functioning, maltreatment, resilience, protective factor

## Abstract

Exposure to childhood victimisation (i.e. abuse, neglect, domestic violence or bullying) can detrimentally impact later psychosocial adjustment. However, this is not the case for all victimised children; some do well despite their experiences and are considered to be resilient. Understanding the factors associated with such resilience is important to inform interventions to support better psychosocial outcomes among victimised children. This review provides an overview of the extant research examining resilience factors for psychosocial outcomes during the transition to adulthood following exposure to childhood victimisation. Studies were identified through a systematic literature search of Embase, PsychINFO and Ovid MEDLINE databases. The 26 included studies spanned a range of psychosocial outcomes between ages 18–25, including education and work, housing and independent living, criminal behaviour, victimisation, and social and psychological adjustment. For each outcome, a variety of putative resilience factors had been investigated including those related to the individual, their family and the wider community within which they lived. However, because few studies had comparable resilience factors and psychosocial outcomes, it is difficult to draw conclusions about which factors are consistently associated with resilience to a particular psychosocial outcome. Additionally, this review revealed that the included studies were of variable methodological quality – many were limited by cross-sectional designs with retrospective self-reports of childhood victimisation, and convenience or unrepresentative samples. In this review, we also highlight gaps in knowledge about the co-occurring impact of multiple resilience factors in combination and the need for studies conducted in non-Western and low- and middle-income countries.

Childhood victimisation – that is, exposure to abuse, neglect, domestic violence or bullying – affects the lives of millions of children around the world (Stoltenborgh et al., 2015). These experiences are distressing for the child at the time and can also have negative long-term impacts including for psychosocial outcomes. For example, victimised children are more likely than their non-victimised peers to have lower educational attainment (Currie & Widom, 2010) and life satisfaction (Fergusson, McLeod, & Horwood, 2013), to be unemployed (Brimblecombe et al., 2018), involved in criminal offending (Malvaso, Delfabbro & Day, 2018) and to perpetrate abuse (Ben-David, Jonson-Reid, Drake, & Kohl, 2015). Moreover, children who are exposed to one type of victimisation commonly also experience other types – termed ‘poly-victimisation’ (Turner, Finkelhor, & Ormrod, 2010) – which further adversely impacts later psychosocial functioning (Wemmers et al., 2018).

Although there has been much focus on the negative impacts of childhood victimisation, not all children with these experiences go on to have such poor psychosocial outcomes; some do well and are therefore considered to be ‘resilient’ (McGloin & Widom, 2001). For instance, in a large UK cohort Latham et al. (2019) found that almost 53% of children exposed to victimisation had no adverse economic outcomes at age 18 and 37% had no adverse psychosocial outcomes. As others have noted, there is no single agreed way to operationalise resilience (Klika & Herrenkohl, 2013; Yoon et al., 2021) – for example, whether resilience is signified by the absence of a poor outcome, the presence of an exceptional outcome or an outcome that is normative despite exposure to victimisation is a discussion beyond the scope of this review. However, there is a broad consensus regarding the concept of resilience as being inferred on the basis of a desirable outcome that occurs in the context of exposure to significant adversity (Rutter, 2006). Thus, resilience is a dynamic process of adaptation rather than an internal, relatively fixed construct or trait that is directly measurable (Luthar, Cicchetti, & Becker, 2000).

This resilience framework provides a useful means to understand why some children are resistant to the negative effects of their victimisation exposure. Factors may be associated with resilience following childhood victimisation via (i) a direct relationship (main effect) that increases positive outcomes or decreases poor outcomes or (ii) they may buffer the negative effect of the childhood victimisation (interaction effect). The identification of such ‘promotive’ and ‘protective’ factors (collectively referred to here as ‘resilience factors’) is important to illuminate potential mechanisms and targets for interventions to support better psychosocial outcomes among victimised children.

Existing systematic reviews of resilience factors have focussed on maltreatment (i.e. abuse, neglect and domestic violence) as the childhood victimisation exposure of interest (Afifi & MacMillan, 2011; Meng et al., 2018; Klika & Herrenkohl, 2013; Fogarty et al., 2019; Yoon et al., 2021). Moreover, none of these have focussed specifically on psychosocial outcomes therefore the extent of research in this domain and the resilience factors that have been identified for psychosocial adjustment is unclear. Importantly, most of these existing reviews have included studies across the lifespan and have typically considered resilience within childhood, adolescence and adulthood which has highlighted developmental variations in resilience factors (Yoon et al., 2021). However, this focus on discrete periods ignores developmental transitions that occur in between. The transition from adolescence to young adulthood is particularly significant: in industrialised countries this previously short transition is now a much-extended period due to demographic changes including continued education and delayed marriage and parenthood (Arnett, 2000). Commonly regarded as lasting from the late teens to mid-twenties (roughly 18–25 years), the transition to adulthood – also termed ‘emerging adulthood’ – is recognised as distinct from adolescence and adulthood (Arnett, 2007). Developmentally salient tasks include exploring one’s identity and role, establishing social connections and romantic relationships, developing independent living skills, securing suitable housing and entering the labour market. It is, therefore, a critical period for resilient psychosocial functioning in order to lay the foundations for success as an adult.

The transition to adulthood is especially prominent for individuals exposed to childhood victimisation who live in out-of-home placements (e.g. foster and residential care) as they are emancipated from – or ‘age out’ of – the care system, usually around the age of 18. Unlike those who live at home, relying on family support while they gradually gain independence is often not an option that is available to those leaving care. Seeking to fill this gap are policies of extended care beyond the age of 18 and programmes to support the leaving care transition in some countries and US states. Nevertheless, navigating the transition to adulthood in the context of leaving care is especially challenging. Understanding factors associated with psychosocial resilience during this transition is therefore critical.

## The Current Review

To address the identified knowledge gaps and augment existing work, our systematic review focusses on factors associated with resilience to psychosocial outcomes during the transition to adulthood. Consistent with evidence on poly-victimisation, whereby different forms of victimisation are found to frequently co-occur (Fisher et al., 2015; Turner et al., 2010), we do not focus on exposure to a single type of childhood victimisation. Instead, we examine resilience following childhood victimisation more broadly, including exposure to abuse, neglect, domestic violence as well as peer victimisation thereby expanding the scope of previous reviews. Furthermore, given the developmental tasks prominent during the transition to adulthood and recommendations to consider resilience in developmentally relevant ways (Yoon et al., 2021), we focus specifically on psychosocial outcomes during the transition to adulthood with the aim of understanding (i) the range of outcomes that have been investigated, (ii) the resilience factors that have been investigated and found to be associated with psychosocial outcomes and (iii) the methodological quality of the studies. This will provide important insights for future research, policy and interventions to help these victimised individuals prosper during the critical transition to adulthood period.

## Method

### Search Strategy and Selection Criteria

An electronic search was conducted of Embase, PsychINFO and Ovid MEDLINE databases for peer-reviewed journal articles written in English and published before December 02, 2019, that examined resilience factors associated with psychosocial outcomes during the transition to adulthood following childhood victimisation (See Appendix A). We searched abstracts and titles using the following terms: ‘child* maltreat*’ OR ‘physical abuse’ OR ‘battered child’ OR ‘child* abuse’ OR ‘negligent treatment’ OR ‘emotional abuse’ OR ‘sexual abuse’ OR molest* OR ‘psychological abuse’ OR neglect OR ‘child victim*’ OR bullying OR bullied OR ‘domestic violence’ OR ‘family violence’ OR ‘interparental violence’ OR ‘inter-parental violence’ OR ‘intimate partner violence’ OR ‘partner abuse’ OR ‘child* trauma’ OR ‘child* advers*’ OR ‘child* exploit*’ OR ‘child welfare’ OR ‘authority care’ OR foster OR adopt OR ‘out-of-home care’ AND resilien* OR protect* OR ‘successful adaptation’ OR coping OR invulnerab* OR prevent* OR hardiness OR positive OR promot*. We did not limit our search by outcome. Reference lists of included papers were hand-searched to identify additional relevant articles. The inclusion and exclusion criteria used to select articles for this review are detailed in Table 1.

**Table 1.** Inclusion and Exclusion Criteria.

	Inclusion Criteria	Exclusion Criteria
Exposure	Assess victimisation (abuse, neglect, bullying or domestic violence) that occurred during childhood (defined as < 18 years old)	Assess victimisation that only occurred in adulthood (> 18 years) or where it is not possible to separate out the childhood exposure from adult exposure
		Assess childhood victimisation as part of a broader childhood adversity or trauma score (e.g. ACE score) which includes experiences other than victimisation, and childhood victimisation is not separated out in the analyses (e.g. by subgroup analysis)
	If the study does not include childhood victimisation as an independent variable but conducts analysis within a sample of former foster youth/youth leaving care, there is information to indicate that they had all experienced childhood victimisation or ≥ 80% of the sample had experienced childhood victimisation	Study does not include childhood victimisation as an independent variable but conducts analysis within a sample of former foster youth/youth leaving care with no information to indicate that they had experienced childhood victimisation or < 80% of the sample had experienced childhood victimisation
Outcome	Yield outcome data in the psychosocial/functional domain	Assess psychosocial/functional outcomes in combination with other non-psychosocial outcomes (e.g. psychopathology and physical health), and it is not possible to separate out the psychosocial outcomes in the analysis
	Psychosocial outcome is assessed for all participants in the transition to adulthood, defined as 18–25 years old, or it is possible to separate out just the transition to adulthood outcomes in the analysis. Where information regarding the participants’ age range is not available, the mean age of the sample falls within 18–25 years	Assess psychosocial/functional outcomes only in childhood (< 18 years old) or later adulthood (> 25 years old) or where it is not possible to separate out just the transition to adulthood outcomes
Study design	Quantitative observation study or qualitative investigation of factor(s) that are associated with the presence of resilience (defined as a positive outcome following childhood victimisation) or clearly and deliberately investigate factor(s) that reduce the risk of a poor outcome following childhood victimisation	Define a resilient outcome as a trait of the individual that is measured on a scale rather than as something which is inferred from positive outcomes despite adverse experience Assess only the prevalence or likelihood of resilience/positive outcome with no investigation of factors associated with it Describe or evaluate the effectiveness of an intervention
Publication type	Published in English in a peer-reviewed journal	Dissertation, conference abstract or do not report primary research (e.g. review paper, commentary, editorial and opinion piece)

*Note.* ACE = adverse childhood experience.

### Data Analysis

Identified titles and abstracts were screened by one researcher (RML). Two researchers (RML and JBN) independently screened full texts and any inconsistencies were resolved through discussion with a third researcher (HLF). Data extracted included author names, date of publication and country of study as well as information related to sample size and selection; participant characteristics and retention; study design; childhood victimisation exposure; measurement of victimisation, resilience factor(s), and outcome(s); analytic approach; and key findings. The methodological quality of included quantitative studies was assessed by RML in consultation with HLF using an adapted version of the Newcastle–Ottawa Scale for cohort studies (Wells et al., 2003), which is recommended by the Cochrane Collaboration (Higgins & Green, 2011). This entailed 11 criteria (scored Yes = 1; No/Not Applicable = 0; see Appendix B) with higher scores reflecting higher methodological quality.

## Results

**Figure 1.**
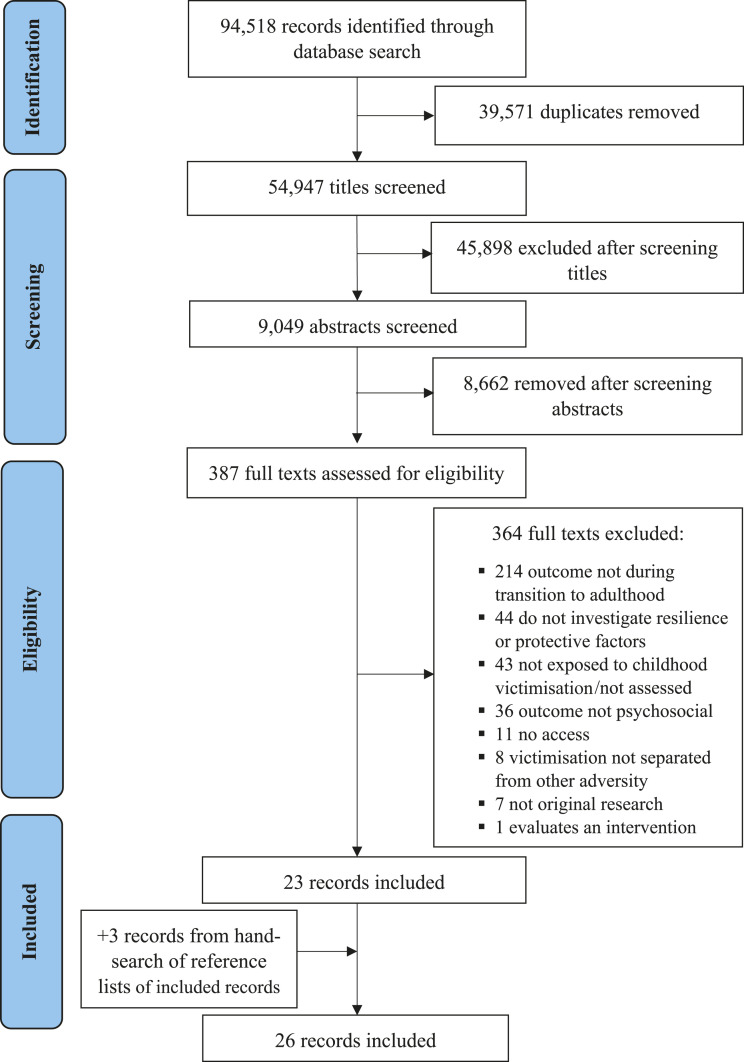
PRISMA (Preferred Reporting Items for Systematic Reviews and Meta-Analyses) flowchart of literature selection.

As shown in Figure 1, our database search yielded 94,518 records. After removing duplicates, screening titles and abstracts, and assessing full-text eligibility, 23 records were retained. A hand-search of these reference lists yielded an additional 3 records. Thus, a total of 26 studies (involving 26 independent samples) were included in this review.

### Characteristics of Included Studies

Included studies were qualitative (*N* = 1) and quantitative (*N* = 25) with cross-sectional (*N* = 13) and longitudinal (*N* = 13) designs. They were published between 2005 and 2019 with 85% published since 2011. The majority of the studies (58%) were conducted in the United States and, with the exception of one study in China, all were conducted in Western, educated, industrialised, rich and democratic (WEIRD) countries. The assessed methodological quality of the quantitative studies ranged from a score of one to nine out of 11, with a median score of four. The quality assessment score for each quantitative study is shown in Table 2 with full details provided in Appendix C.

**Table 2.** Summary of Studies Included in the Review.

Author, year	Country	Study Design	Sample	Childhood Victimisation	Resilience Factor(s)	Outcome(s)	Key Relevant Finding(s)	QA
Abajobir et al. (2017)	Australia	Long	*N *= 3818 children followed up (53% female)	Maltreatment (sexual, physical, emotional abuse and neglect)	Sex	Delinquency	None of the childhood maltreatment types increased the risk of delinquency for females; but for males, any type except sexual abuse increased delinquency risk	8
Beduna & Perrone-McGovern (2019)	USA	Cross	*N* = 322 University students (76% female)	Bullying	Self-compassion and emotional regulation	Shame	Bullying associated with shame via its negative association with self-compassion (which in turn increased shame). No indirect association via emotion regulation as bullying not related to this	3
Bonanno et al. (2007)	USA	Long	*N* = 115 (54 abused and 61 non-abused comparison) (100% female)	Sexual abuse	Genuine smiling and laughter and non-genuine smiling while describing a distressing event of their choice	Social adjustment	Genuine positive emotional expression is beneficial for social adjustment only when expressed in appropriate contexts (i.e. not when describing a past abuse experience)	8
Brendgen & Poulin (2018)	Canada	Long	*N* = 251 children followed up (60% female)	Bullying	Peer social support	Work-place victimisation	Peer social support offset the indirect effect of bullying on work-place victimisation by reducing depressive symptoms	4
Edwards et al. (2014)	USA	Cross	*N* = 765 university students (100% female)	Maltreatment (sexual, physical, and verbal abuse, and witnessing IPV	Resiliency characteristics (e.g. can handle unpleasant feelings, thinks of self as strong person)	Interpersonal distress	Resiliency*maltreatment interaction not significant – resiliency characteristics associated with lower levels of interpersonal distress, but maltreatment still has adverse effect on interpersonal distress even when resiliency characteristics are high	3
Fagan (2005)	USA	Long	*N* = 1725 adolescents followed up (47% female)	Physical abuse by parent(s)	Sex, ethnicity, family structure, urbanicity of residence and family income	Criminal offending (prevalence and frequency)	Adverse impact of abuse on offending frequency was weaker for higher incomes and suburban (vs. urban) areas. Adverse impact of abuse on frequency of minor partner violence was weaker for those with 2 biological parents (vs. broken home). Adverse impact of abuse on serious partner violence frequency was weaker for higher incomes, 2 biological parents (vs. broken home) and rural (vs. suburban) areas	9
Fallesen (2013)	Denmark	Long	*N* = 7220 individuals formerly in foster care (% female unclear)	Maltreatment	Duration of time in foster care	Education attainment, social assistance dependency, unemployment and income	More time in foster care associated with higher income and lower unemployment. No association with education attainment or social assistance dependency	7
Folger & Wright (2013)	USA	Cross	*N* = 344 university students (54% female)	Maltreatment (physical, sexual, emotional abuse and physical neglect)	Gender, perceived social support from family and friends	Received physical and emotional dating abuse	Perceived family support was a protective factor for dating abuse when exposure to childhood maltreatment was low but a vulnerability factor when exposure was high	3
Fowler et al. (2017)	USA	Long	*N* = 350 children subject to child abuse and neglect investigations followed up (62% female)	Abuse and neglect	Age-18 ageing out of care status, state policy of extended foster care beyond age 18 and receipt of independent living services	Homelessness and housing instability	Those who reunified with family were less likely to experience homelessness than those who aged-out of care or were never placed out of home. Aged-out and never placed individuals had similar levels of homelessness. No group differences on prevalence of unstable housing. Exposure to independent living services and extended foster policies were un-related to homelessness	6
Galea et al. (2007)	Malta	Cross	*N* = 312 sixth form and university students (69% female)	Abuse and neglect	Spirituality and religious practices	Wellbeing (satisfaction with life, positive affect and negative affect)	Positive association of spirituality (but not religious practices) with positive affect and life satisfaction regardless of exposure to childhood abuse and neglect	3
Jaffee et al. (2018)	UK	Long	*N* = 2066 children followed up (47% female)	Physical and sexual maltreatment	Supportive adult involvement	Educational attainment, NEET status	Higher levels of adult involvement associated with better education attainment whether exposed to maltreatment or not. Supportive adult involvement did not moderate impact of maltreatment on educational attainment or NEET status	9
Kalaitzaki (2019)	Greece	Cross	*N* = 807 university students (72% female)	Receiving punitive discipline, witnessing mutual IPV	Closeness to mother and father	Involvement in mutual dating violence	Witnessing IPV associated with lower levels of closeness to mother which in turn was associated with a lower probability of mutual dating violence. Punitive discipline was not associated with closeness to mother	4
Lane (2017)	USA	Cross (Qual)	*N* = 10 African American university students formerly in foster care (80% female)	Abuse or neglect	(Not pre-determined)	College/University enrolment	Enrolment was influenced by wanting to rise above their circumstances/avoid repeating the cycle of family detriments and to prove wrong those with low expectations of them. College degree viewed as a route to broader success	NA
Latham et al. (2019)	UK	Long	*N* = 506 (53% female) for psychosocial disadvantage outcome. *N* = 503 (52% female) for economic disadvantage outcome Children within a birth cohort exposed to victimisation followed up	Abuse, neglect, exposure to domestic violence and bullying	IQ, SES, maternal warmth, personality, number of biological parents in household, sibling warmth, adult involvement, social cohesion, status among peers, low neighbourhood crime victimisation, social cohesion and sex	Any psychosocial disadvantage (presence of 1+ of poly-victimisation, low life satisfaction, social isolation, loneliness, low sleep quality) Economic disadvantage (presence of 1+ of low educational achievement, NEET status, parenthood criminal cautions and convictions)	Resilience to psychosocial disadvantage best predicted by a combination of being male; lower anxiety, depression and CD symptoms; lower self-harm/suicide; higher maternal and sibling warmth; higher adult involvement; lower family history of psychopathology; lower neighbourhood crime victimisation; higher social cohesion and higher status among peers. Resilience to economic disadvantage best predicted by a combination of being female; higher IQ; higher consciousness, extraversion, agreeableness, and neuroticism; lower ADHD symptoms; higher maternal and sibling warmth; and higher SES.	6.5
Lee et al. (2012)	USA	Long	*N* = 729 individuals transitioning out of care (52% female)	Abuse or neglect	Care status beyond age 18, education, employment, family formation	Criminal behaviour (violent crime, property crime, drug selling or any crime) Involvement in the criminal legal system (arrest, incarceration conviction)	Care status did not predict criminal behaviour. For males, employment reduced likelihood of property and drug crime; a high school diploma reduced odds of property crime; a resident child reduced likelihood of drug and violent crime. None reduced odds of criminal behaviour for females Being in care at the previous interview did not predict legal involvement for males but reduced the odds of arrest for females. Having a child reduced likelihood of arrest for males and arrest and incarceration for females For males, being enrolled at school lowered the odds of arrest, a high school diploma lowered odds of arrest and incarceration	6
Liu et al. (2018)	China	Cross	*N* = 554 university students (77% female)	Emotional abuse	Secure attachment	Self-esteem	Emotional abuse negatively associated with secure attachment which, in turn, was positively associated with self-esteem	3
Maples et al. (2014)	USA	Cross	*N* = 301 university students (54% female)	Abuse and neglect	Sex, social and emotional resources (intelligence, positive caregiving, good schools, parental expectations, self-esteem, talent, faith, family connectedness, financial resources)	College adjustment	More males were well-adjusted than females in maltreated group. Good schools and self-esteem associated with better college adjustment. For females, the negative association between maltreatment and college adjustment was partially mediated by negative life events and fully mediated by social and emotional resources	1
Miller-Graff et al. (2016)	Sweden	Cross	*N* = 703 individuals exposed to IPV (58% female)	IPV exposure	Mother–child warmth, father–child warmth	Life satisfaction	Negative association between IPV exposure and lower life satisfaction fully explained by the negative association of IPV with mother- and father-child warmth	3
Mohr & Rosén (2017)	USA	Cross	*N* = 501 university students (72% female)	Abuse (physical, sexual and emotional) and neglect	Social and emotional resources (self-esteem, acceptance, presence of pro-social adults and emotional support, optimism, positive reframing)	Post-traumatic growth (PTG)	Acceptance, emotional support, and positive reframing predicted PTG. Maltreatment was positively associated with PTG when presence of pro-social adults was high but no association when it was low. Positive association between maltreatment and PTG when individuals had a high number of social and emotional resources, but no relationship when resources were low	2
Oshri et al. (2018)	USA	Long	*N* = 1461 children subject to maltreatment investigations and followed up (56% female)	Maltreatment	Future orientation (3 groups: High-persistent, low start/increasing and high start/decreasing)	Arrests, developmental milestones (independent living skills, social support and capital, income and employment)	High-persistent and low start/increasing future orientation groups were more likely to attain developmental milestones	6
Paat & Markham (2019)	USA	Cross	*N* = 3495 university students (70% female)	Neglect, harsh corporal punishment, witnessing DV	Residing in a two-parent household, parental education, and commitment to the dating relationship	Physical aggression in dating relationship (received and perpetrated)	Two-parent household negatively associated with received and perpetrated physical aggression in dating relationship whether exposed to childhood victimisation or not (resilience factors did not moderate impact of victimisation on dating aggression)	3
Pepin & Banyard (2006)	USA	Cross	*N* = 202 university students (68% female)	Physical, psychological& sexual abuse, and neglect	Perceived social support from family and friends	Developmental achievement (trust, autonomy, initiative, industry, identity and intimacy)	Perceived social support mediated association between maltreatment and developmental achievement	4
Schaefer et al. (2018)	USA	Cross	*N* = 161 university students exposed to physical and/or sexual abuse (86% female)	Physical, sexual abuse	Social support (family and friendship connections), optimism, religious coping	PTG	Optimism and positive religious coping were associated with higher PTG	2
Strøm et al. (2013)	Norway	Long	*N* = 11,874 adolescents followed up (51% female)	Physical violence, sexual abuse, bullying	High school completion within 5 years	Work participation	High school completion reduced adverse impact of violence and bullying on work participation, but the association remained significant	8
Tracy et al. (2018)	UK	Long	*N* = 11,384 children followed up (48% female)	Maltreatment (emotional, physical, or sexual)	Paternal involvement	Physical violence perpetration	Paternal involvement reduced the risk of physical violence, but maltreated individuals remained at higher risk of physical violence perpetration, especially if it began in early or middle childhood. The general protective role of paternal involvement was evident for both sexes, regardless of maltreatment exposure, type or timing	6
Tyrell & Yates (2017)	USA	Mixed. (Long for most, cross for 32% of sample)	*N* = 172 maltreated individuals transitioning out of care (66% female)	Abuse (physical, sexual and emotional), neglect and exposure to domestic violence	Age at emancipation, female sex, parenting status, Europe American ethno-racial status and educational attainment	Housing quality after emancipation (level and change in housing quality)	Being a parent and having a high school degree associated with better housing quality 6 months after emancipation	3.5

*Note.* QA = methodological quality assessment (scored out of 11; higher scores reflect higher quality); Cross = cross-sectional study design; Long = longitudinal study design; Qual = qualitative study; IPV = intimate partner/inter-parental violence; NEET = Not in Education, Employment or Training; ADHD = attention deficit hyperactivity disorder; SES = socio-economic status; PTG = post-traumatic growth.

The included studies examined resilience factors associated with psychosocial outcomes related to education and work (e.g. educational attainment; work participation; Not in Education, Employment, or Training (NEET) status; income; social assistance dependency), housing and independent living (e.g. housing problems, housing quality and independent living skills), criminal behaviour (e.g. delinquency and criminal offending), victimisation (e.g. work-place victimisation, perpetrated and/or received dating abuse), social outcomes (e.g. social support, social adjustment and interpersonal distress) and psychological outcomes (e.g. life satisfaction, shame, self-esteem and post-traumatic growth). The evidence regarding factors associated with resilience in each of these psychosocial areas is summarised below.

### Evidence Overview and Synthesis

*Education and work.* Six studies explored factors related to resilience in education and work following exposure to childhood maltreatment (Fallesen, 2013; Jaffee et al., 2018; Lane, 2017; Maples, Park, Nolen, & Rosén, 2014; Oshri, Duprey, Kogan, Carlson, & Liu, 2018; Strøm et al., 2013) or bullying (Strøm et al., 2013). Five of the studies were quantitative with all but one (Maples et al., 2014) using longitudinal designs. Three studies conducted analyses within samples of individuals exposed to childhood victimisation. This included those identified from population registers who had spent time in foster care (Fallesen, 2013), university students who were formerly in foster care (Lane, 2017) and individuals who had been the subject of maltreatment investigations during childhood (Oshri et al., 2018). Two other studies used general population-based samples – one measured maltreatment exposure prospectively (Jaffee et al., 2018), the other used retrospective self-reports of exposure to violence, abuse and bullying (Strøm et al., 2013). Lastly, one study used a convenience sample of university students who retrospectively reported experiences of childhood maltreatment (Maples et al., 2014). The assessed quality of the quantitative studies ranged from a score of one (Maples et al., 2014) to nine (Jaffee et al., 2018) out of 11.

Taken together, quantitative findings demonstrated a positive effect of increased duration of foster care (Fallesen, 2013) and having a future orientation (i.e. expectations of achieving adult milestones; Oshri et al., 2018) on income and employment at the transition to adulthood; a protective role of being male, attending a good school and having higher self-esteem on college adjustment (Maples et al., 2014); and higher levels of supportive adult involvement on educational attainment for maltreated and non-maltreated individuals alike (Jaffee et al., 2018). Furthermore, high school completion was found to partially reduce the negative impact of exposure to childhood bullying and physical violence on work participation. However, even those exposed individuals who completed high school remained at a significantly higher risk of not participating in work during the transition to adulthood than their non-victimised peers (Strøm et al., 2013).

Qualitative findings identified that among a sample of African American young adults who were formerly in foster care the decision to enrol at college/university was motivated by a desire to rise above their circumstances to avoid repeating the cycle of family detriments as well as feeling like they had something to prove to their family and/or foster parents (Lane, 2017). Achieving a college education was seen as being the means to a better life and these individuals’ childhood experiences were key motivating factors.

*Housing and independent living.* Studies were identified that examined factors associated with better housing outcomes (*n* = 2) and independent living skills (*n* = 1) during the transition to adulthood following exposure to childhood maltreatment (Fowler, Marcal, Zhang, Day, & Landsverk, 2017; Oshri et al., 2018; Tyrell & Yates, 2017). All three studies were longitudinal and conducted in the USA with individuals who were either transitioning out of the care system or had been subject to child maltreatment investigations but were not placed out of home. The methodological quality of the studies was assessed as three-and-a-half (Tyrell & Yates, 2017) and six (Fowler et al., 2017; Oshri et al., 2018) out of 11.

Findings of Fowler et al. (2017) suggest that individuals who were placed in care but reunified with their family by the age of 18 were significantly less likely to experience homelessness than those who either ‘aged out’ of care or who remained living at home. Notably, exposure to independent living services and a state policy of extended foster care were not found to be associated with homelessness prevention. Moreover, individuals who were parents and those with higher educational attainment were found to have better quality housing 6 months after emancipation from foster care (Tyrell & Yates, 2017).

Individuals’ future orientation was found to be important for independent living skills during the transition to adulthood following childhood maltreatment (Oshri et al., 2018). In particular, those who initially scored low on future orientation but who showed an increase in this during adolescence were found to subsequently have more independent living skills than those who scored persistently high on future orientation.

*Criminal behaviour.* Four studies examined factors associated with resilience for criminal outcomes, three following exposure to childhood maltreatment (Abajobir et al., 2017; Lee et al., 2012; Oshri et al., 2018) and one following physical abuse specifically (Fagan, 2005). These studies were all quantitative and longitudinal; one used a sample who were transitioning out of care (Lee et al., 2012), one followed up a sample of children who had been subject to maltreatment investigations (Oshri et al., 2018) and the other two studies measured maltreatment and physical abuse exposure, respectively, within general population samples of children and adolescents who were followed to young adulthood (Abajobir et al., 2017; Fagan, 2005). The assessed methodological quality of the studies ranged from six (Lee et al., 2012; Oshri et al., 2018) to nine (Fagan, 2005) out of 11.

Demographic factors were examined as potential resilience factors by three studies. Contradictory findings were evident with regards to the moderating role of sex: On the one hand, documented childhood maltreatment was found not to increase the risk of self-reported delinquency for females, whereas it did for males (Abajobir et al., 2017). However, the association between physical abuse during adolescence and the prevalence of criminal offending was not moderated by sex (Fagan, 2005), suggesting that different victimisation exposures may interact differently with sex in relation to criminal behaviour. Instead of sex, in this general population sample, family and neighbourhood factors were found to be important such that the link between physical abuse and criminal offending prevalence was weaker for those in suburban and rural rather than urban neighbourhoods, those from higher income families, and those from two-parent families (Fagan, 2005). Among individuals transitioning out of care, being employed, achieving a high school diploma and having a resident child lowered the odds of criminal behaviour and legal system involvement for males, and remaining in care lowered the odds for legal system involvement for females (Lee et al., 2012). Finally, one study compared the number of arrests according to individuals’ level of future orientation during adolescence but found no differences (Oshri et al., 2018).

*Victimisation (received and/or perpetrated).* We identified five quantitative studies that examined resilience factors associated with received and/or perpetrated victimisation during the transition to adulthood (Brendgen & Poulin, 2018; Folger & Wright, 2013; Kalaitzaki, 2019; Paat & Markham, 2019; Tracy, Salo, & Appleton, 2018). Three studies were cross-sectional and used convenience samples of university students, and two studies were longitudinal and followed children into young adulthood. The methodological quality of the studies was assessed as ranging from a score of three (Folger & Wright 2013; Paat & Markham, 2019) to six (Tracey et al., 2018) out of 11.

Studies that focussed on dating relationship abuse following exposure to childhood maltreatment and/or witnessing inter-parental violence (*n* = 3) examined the potential beneficial effect of a two-parent family structure and positive relationships with family and friends. Having two married parents was found to be associated with lower concurrent levels of received and perpetrated physical aggression in dating relationships for victimised and non-victimised individuals alike (Paat & Markhan, 2019). Findings regarding the role of family relationships were more complex: perceived social support from family was found to be protective against women’s received dating abuse when their exposure to childhood maltreatment was low. However, when their maltreatment exposure was high, perceived social support from family increased their vulnerability for receiving dating abuse suggesting that it may not be a resilience factor for women exposed to severe childhood maltreatment (Folger & Wright, 2013). Moreover, those exposed to mutual inter-parental violence in childhood were found to be less likely to engage in mutual dating violence during the transition to adulthood if they concurrently reported feeling less closeness with their mother (Kalaitzaki, 2019), contrary to the often-assumed general benefit of close parent–child relationships.

In contrast, a longitudinal study of children followed into young adulthood found that more frequent and more positive father–child interactions reduced the risk of later physical violence perpetration following childhood maltreatment, though this was not specific to violence in dating relationships (Tracy et al., 2018). Despite this reduction in risk, however, even those exposed to maltreatment who had high levels of father involvement continued to be at an increased risk of violence compared to their non-maltreated peers (Tracy et al., 2018). The role of positive parent–child relationships and family support is therefore mixed and may depend on the specific type of childhood victimisation (e.g. maltreatment vs. inter-parental violence), the severity of this, and the particular outcome of interest (e.g. dating abuse vs. general violence).

Finally, one study focussed on childhood exposure to bullying and being victimised at work during the transition to adulthood (Brendgen & Poulin, 2018). The indirect effect of bullying on workplace victimisation via depressive symptoms was found to be counteracted by social support from friends during adolescence.

*Social outcomes.* Three quantitative studies focussed on resilience to social outcomes including social support and capital (Oshri et al., 2018), interpersonal distress (Edwards, Probst, Rodenhizer-Stämpfli, Gidycz, & Tansill, 2014) and social adjustment (Bonnano et al., 2007) following childhood maltreatment. Studies were cross-sectional (*n* = 1), retrospectively measuring abuse and neglect among university students and longitudinal (*n* = 2), either using a sample of individuals who had been the subject of child maltreatment investigations or comparing females exposed to child sexual abuse with a non-abused comparison group. The assessed quality of the studies ranged from three (Edwards et al., 2014) to eight (Bonanno et al., 2007) out of 11.

Two studies examined personal characteristics as potential resilience factors for social outcomes following childhood maltreatment. Resiliency characteristics (e.g. thinks of self as strong person and tends to bounce back after illness or hardship) were found to be concurrently associated with lower levels of interpersonal distress in a sample of female university students (Edwards et al., 2014). However, even when resiliency characteristics were high, maltreatment still exerted a significant influence on interpersonal distress (Edwards et al., 2014). In another study, being future orientated was found to promote more social support and capital among those exposed to childhood maltreatment (Oshri et al., 2018).

The expression of genuine positive emotion (e.g. genuine smiles and laughter) was examined by the third study (Bonnano et al., 2007). This was found to be beneficial for social adjustment when expressed in appropriate but not inappropriate contexts. For example, child sexual abuse survivors who expressed genuine positive emotion while describing a past abuse experience were found to have poorer social adjustment (Bonnano et al., 2007).

*Psychological outcomes.* We identified six studies that examined resilience factors associated with psychological outcomes including post-traumatic growth (positive psychological change as a result of a challenging experience) (PTG; Mohr & Rosén, 2017; Schaefer, Howell, Schwartz, Bottomley, & Crossnine, 2018), feelings of shame (Beduna & Perrone-McGovern, 2019), self-esteem (Liu et al., 2018), and positive and negative affect (Galea, Ciarrocchi, Piedmont, & Wicks, 2007). Studies were quantitative and employed cross-sectional designs in which participants retrospectively reported their childhood victimisation experiences. One study used a sample that was randomly selected from a national inhabitant register (Miller-Graff, Cater, Howell, & Graham-Bermann, 2016), whereas all others used convenience samples of university students. The methodological quality of the identified studies ranged from two to three out of 11.

Two studies examined life satisfaction during the transition to adulthood – one following childhood exposure to intimate partner violence (IPV; Miller-Graff et al., 2016) and the other following abuse and neglect (Galea et al., 2007). The detrimental impact of witnessing IPV during childhood on later life satisfaction was found to be explained by its negative impact on parent–child relationship warmth. That parent–child warmth was itself positively associated with life satisfaction suggests it may be a key resilience factor (Miller-Graff et al., 2016). Furthermore, spirituality (but not religious practices) was found to be positively associated with life satisfaction and positive affect whether individuals were exposed to childhood abuse and neglect or not, suggesting the beneficial effects of spirituality extend to those with such adverse childhood experiences (Galea et al., 2007).

In two other studies of individuals who experienced childhood abuse and/or neglect, optimism, religious coping (e.g. religious forgiving and seeking spiritual support), positive reframing, acceptance and emotional support were found to be associated with higher levels of PTG at the transition to adulthood (Mohr & Rosen, 2017; Schaefer et al., 2018). Furthermore, the presence of a pro-social adult and having a higher number of social and emotional resources overall (e.g. intelligence, self-esteem and good school) moderated the relationship between maltreatment and PTG such that childhood exposure to maltreatment was positively associated with PTG when these resilience factors were high (Mohr & Rosen, 2017). One other study found that secure attachment mediated the relationship between childhood emotional abuse and self-esteem (Liu et al., 2018), in that emotional abuse negatively affected secure attachment which was itself positively associated with self-esteem.

Lastly, childhood bullying was found to be related to feelings of shame during the transition to adulthood via reduced feeling of self-compassion suggesting that promoting self-compassion following exposure to bullying may protect against subsequent feelings of shame (Beduna & Perrone-McGovern, 2019).

*Multi-domain psychosocial outcomes.* Two quantitative studies examined multiple psychosocial outcomes in combination; one was cross-sectional and used a sample of university students’ retrospective reports of maltreatment (Pepin & Banyard, 2006) and the other was longitudinal and used prospectively measured exposure to maltreatment, bullying and domestic violence (Latham et al., 2019). The methodological quality of the studies was assessed as four and six-and-a-half, respectively.

Perceived social support from friends and family was found to explain the relationship between childhood maltreatment and the achievement of Erikson’s stages of psychosocial development (Erikson, 1968), suggesting that this is important in shaping psychosocial development among university students who have experienced childhood maltreatment (Pepin & Banyard, 2006). An examination of multiple factors related to the individual, family and community found that a different combination was needed to best predict whether or not a victimised individual would have poor psychosocial or economic outcomes during the transition to adulthood (Latham et al., 2019). Psychosocial resilience was best predicted by a combination of being male, having lower levels of anxiety, depression and conduct disorder symptoms; lower levels of self-harm/suicide attempts; higher maternal and sibling warmth; higher levels of adult involvement; lower family history of psychopathology; lower neighbourhood crime victimisation; higher social cohesion and higher status among peers. Economic resilience at the transition to adulthood was best predicted by a combination of being female, having higher intelligence, being more conscientious, extravert, agreeable, and neurotic, having lower levels of ADHD symptoms, higher maternal and sibling warmth, and higher family SES (Latham et al., 2019).

## Discussion

Our review reveals a relatively small research literature on resilience to psychosocial outcomes at the transition to adulthood for individuals exposed to childhood victimisation. The 26 included studies spanned a wide range of psychosocial outcomes that encompassed the key developmental tasks associated with this important transitional life stage, for example, housing, independent living, education, employment and social relationships. For each outcome, a variety of different factors had been investigated including those related to the individual (e.g. demographics and care experience/leaving care status), the family (e.g. parent–child relationship, support and family structure) and community (e.g. support from peers, support services and extended-care policies). Whilst there is evidence for some resilience factors for psychosocial functioning during the transition to adulthood following childhood victimisation, the small number of studies with comparable resilience factors and psychosocial outcomes precludes us from drawing firm conclusions about which are consistently associated with resilience to particular psychosocial outcomes. Further research in this area is therefore needed to bolster the evidence base (Table 3 summarises the critical findings of our review).

**Table 3.** Critical Findings.

• 26 studies examining resilience factors for psychosocial outcomes during the transition to adulthood following childhood victimisation were identified
• Studies have investigated psychosocial outcomes related to education and work, housing and independent living, criminal behaviour, victimisation (perpetrated and/or received), and social and psychological adjustment
• Few studies had comparable resilience factors and psychosocial outcomes which limits conclusions about which are consistently associated
• There is a dearth of studies that use samples from non-WEIRD (Western, educated, industrialised, rich and democratic) countries. We identified only 1 out of 26 included studies
• Quantitative studies varied in methodological quality. The median score for assessed quality was 4 out of 11 (range = 1–9 out of 11)
• Many studies were limited by their use of convenience or other non-representative samples and cross-sectional designs with retrospective reports of childhood victimisation
• Very few studies examine multiple co-occurring resilience factors for psychosocial outcomes

More broadly, our review suggests that a resilience factor for one psychosocial outcome may not necessarily be a resilience factor for a different outcome. For example, positive/supportive relationships were often associated with positive outcomes (e.g. educational attainment and life satisfaction) during the transition to adulthood following childhood victimisation; but in the context of severe childhood maltreatment and exposure to domestic violence, close family relationships were associated with increased vulnerability for perpetration and experience of victimisation (Folger & Wright, 2013; Kalaitzaki, 2019). Though we caution that these latter findings were based on cross-sectional study designs which limits conclusions of cause and effect, the potential for the promotion of one resilience factor to inadvertently increase an individual's vulnerability for something else has important implications for those working to support victimised children. Critically, it highlights the need for research to holistically consider the impact of multiple resilience factors in combination to advance our understanding of co-occurring resilience influences to more accurately reflect real-life. However, our review showed that the majority of studies typically examined only one or a few resilience factors at a time, revealing an important knowledge gap.

The methodological quality of the reviewed studies was wide-ranging. The use of prospective longitudinal study designs with a clear temporal order of victimisation, resilience factor(s) and psychosocial outcome(s) are vital to help clarify the direction of observed associations. However, we found that a large proportion of studies in our review relied on cross-sectional designs using individuals’ retrospective reports of childhood victimisation. This has the potential to introduce time-related memory biases such as inaccuracy due to delay (Hardt & Rutter, 2004) and the reconsolidation of victimisation memories following feedback (e.g. being told something was or was not victimisation; Brewin & Andrews, 2017). Retrospective recall of adverse childhood experiences may also be influenced by current mental health and/or psychosocial functioning, thereby potentially introducing biases in the associations observed. Indeed, associations between retrospective self-reports of childhood maltreatment and self-reported psychosocial outcomes at the transition to adulthood have been noted to be stronger compared to prospective reports obtained from caregivers and researchers (Latham et al., 2021). This raises questions regarding the generalisability of findings from studies that used only individuals’ retrospective reports of childhood victimisation, given that the associations may differ for those who do not remember or choose not to disclose maltreatment. Moreover, recent evidence indicates there is only weak overlap between childhood maltreatment reports that are obtained prospectively and retrospectively (Newbury et al., 2018; Baldwin et al., 2019) meaning that these report types identify different groups of maltreated children and are not interchangeable.

An additional methodological concern highlighted by our review was the use of convenience and other non-representative samples. Studies that use methods to achieve a sample that is representative of the target population (e.g. random or stratified sampling) allow us to generalise their findings to this population with greater confidence than studies that simply select from the target population people who are available at the time. This convenience approach biases the sample, undermines its representativeness and, thus, reduces the generalisability of the study findings to the target population. For instance, several studies in our review used samples of university students to examine resilience during the transition to adulthood. Whilst this may be relevant and of value for work focussed on educational resilience following childhood victimisation, it captures a very particular demographic that likely represents individuals who are functioning relatively well. This therefore limits the conclusions that can be drawn from this sample regarding resilience to other (non-educational) psychosocial outcomes.

Finally, our review highlighted the preponderance of research in WEIRD countries. Childhood victimisation and its detrimental psychosocial impacts are a global concern (World Health Organisation, 2020); however, we cannot assume that findings regarding resilience in studies using WEIRD samples will be the same for other countries. In light of cultural differences in the transition to adulthood (Badger, Nelson, & Barry, 2006; Seiter & Nelson, 2011), the factors associated with psychosocial resilience at this developmental stage following childhood victimisation may also differ. It is therefore important to examine factors associated with resilience to psychosocial outcomes across diverse settings including non-Western, and low- and middle-income countries.

### Limitations

These findings should be considered in the context of some important study limitations. We focussed on childhood victimisation defined as exposure to abuse, neglect, domestic violence and bullying, and therefore, we have not included other exposures such as racial victimisation and community violence. Despite our comprehensive search strategy, there may be relevant studies that were not identified or included in the review including unpublished studies and other grey literature. Given the very large number of records identified by our search, we had only one author undertake the initial screening. Our literature search was limited to studies published in English therefore relevant studies that are published in other languages may exist. This latter point is particularly pertinent to our finding of few studies in non-WEIRD samples.

## Conclusion

Despite the noted limitations, this review provides a comprehensive overview of the current literature regarding factors associated with resilience to psychosocial outcomes in the transition to adulthood following exposure to childhood victimisation. We highlight gaps in current knowledge, methodological limitations of existing evidence and the need for future research that examines multiple resilience factors in combination, utilises longitudinal designs, prospectively reported victimisation and samples from diverse contexts.

### Implications of the review for practice, policy and research

- There is evidence that there are a number of resilience factors for psychosocial outcomes during the transition to adulthood following childhood victimisation, suggesting that interventions that target these may successfully boost resilience.
- Although some factors reduce the impact of childhood victimisation on psychosocial outcomes, these individuals may remain at higher risk of poor outcomes than their non-victimised peers.
- Practitioners working with individuals exposed to childhood victimisation should be aware that factors associated with resilience to one psychosocial outcome do not necessarily also promote resilience to a different outcome. It is possible for something (e.g. close parent–child relationships) to be both a resilience factor and a vulnerability factor depending on the victimisation exposure and outcome of interest.
- Most studies examine only one or a few resilience factors. Future research should examine multiple factors in combination to understand the co-occurring resilient influences on psychosocial functioning during the transition to adulthood for those exposed to childhood victimisation.
- Research is needed from low- and middle-income countries to illuminate whether resilience factors differ in these contexts.

## Appendix A.

### Search Strategy History

**Table:**

Step	Search
1	(‘child* maltreat*’ or ‘physical abuse’ or ‘battered child’ or ‘child* abuse’ or ‘negligent treatment’ or ‘emotional abuse’ or ‘sexual abuse’ or molest* or ‘psychological abuse’ or neglect or ‘child victim*’ or bullying or bullied or ‘domestic violence’ or ‘family violence’ or ‘interparental violence’ or ‘inter-parental violence’ or ‘intimate partner violence’ or ‘partner abuse’ or ‘child* trauma’ or ‘child* advers*’ or ‘child* exploit*’ or ‘child welfare’ or ‘authority care’ or foster or adopt or ‘out-of-home care’).ab,ti
2	(resilien* or protect* or ‘successful adaptation’ or coping or invulnerb* or prevent* or hardiness or positive or promot*).ab,ti
3	1 and 2
4	Limit 3 to English language
5	Limit 4 to human
6	Limit 5 to humans [limit not valid in PsychINFO; records were retained]
7	Limit 6 to peer-reviewed journal [Limit not valid in Embase, Ovid MEDLINE(), Ovid MEDLINE(R) Daily Update, Ovid MEDLINE(R) In-Process, Ovid-MEDLINE (R) Publisher; records were retained]

Databases selected within the Ovid database platform: 1. Embase, 2. PsychINFO, 3. OvidMEDLINE(R) and Epub Ahead of Print, In-Process & Other Non-Indexed citations, Daily and Versions(R)

## Appendix B

### Adapted Version of the Newcastle-Ottawa Quality Assessment Scale

**Table:**

Quality item	Yes/No/NA
1. Are the victimised individuals likely to be representative of the target population at baseline?	
2. Are the victimised individuals likely to be representative of the target population at outcome?	
3. Was the non-exposed group drawn from the same community as the exposed group (for continuous measures of exposure, were all participants from the same community)?	
4. Ascertainment of exposure to childhood victimisation was undertaken through official records or validated (i.e. pre-existing/published) measure?	
5. Clear temporal order of events such that assessment of victimisation occurs prior to assessment of outcome and, where relevant, prior to resilience factors?	
6. Comparability between victimised and non-victimised groups was increased by matching or adjusting for one variable?	
7. Comparability between victimised and non-victimised groups was increased by matching or adjusting for two or more variables?	
8. Objective outcomes assessed by official records/documented evidence? Subjective outcomes assessed by validated (i.e. pre-existing/published) measure?	
9. Were at least 70% of subjects followed up (if longitudinal)?	
10. Application of a longitudinal study design?	
11. Were different types of victimisation analysed separately?	
Total score	/11

## Appendix C.

### Quality Assessment of the Included Quantitative Studies.

**Table:**

	Quality Assessment Item											
Study	1. Are the Victimised Individuals Likely to be Representative of Target Population at Baseline?	2. Are the Victimised Individuals Likely to be Representative of Target Population at Outcome?	3. Non-Exposed and Exposed Drawn from Same Community?	4. Exposure to Victimisation Ascertained via Official or Validated Measure?	5. Clear Temporal Order of Events Such That Victimisation Assessment Occurs Prior to Outcome and Prior to Resilience Factors?	6. Comparability of Exposed and Non-exposed Increased by Matching/Adjusting for 1 Variable?	7. Comparability of Exposed and Non-exposed Increased by Matching/Adjusting for 2+ Variables?	8. Objective Outcomes Measured by Official Evidence/Subjective Outcomes by Validated Measures?	9. At least 70% of Subjects Followed Up if Longitudinal?	10. Longitudinal Design?	11. Different Types of Victimisation Analysed Separately?	Total Score (/11)
Abajobir et al. (2017)			✓	✓	✓	✓	✓	✓		✓	✓	8
Beduna & Perrone-McGovern (2019)			✓	✓				✓				3
Bonanno et al. (2007)			✓	✓	✓	✓	✓	✓	✓	✓		8
Brendgen & Poulin (2018)			✓					✓	✓	✓		4
Edwards et al. (2014)			✓	✓				✓				3
Fagan (2005)	✓	✓	✓		✓	✓	✓	✓	✓	✓		9
Fallesen (2013)	✓	✓		✓	✓			✓	✓	✓		7
Folger & Wright (2013)			✓	✓				✓				3
Fowler et al. (2017)	✓	✓		✓	✓			✓	^a^	✓		6
Galea et al. (2007)			✓	✓				✓				3
Jaffee et al. (2018)	✓	✓	✓	✓	✓	✓	✓		✓	✓		9
Kalaitzaki (2019)			✓	✓				✓			✓	4
Latham et al. (2019)	✓	✓		✓	✓			½^b^	✓	✓		6.5
Lee et al. (2012)	✓	✓		✓	✓				✓	✓		6
Liu et al. (2018)			✓	✓				✓				3
Maples et al. (2014)			✓									1
Miller-Graff et al. (2016)	✓	✓		✓								3
Mohr & Rosén (2017)			✓					✓				2
Oshri et al. (2018)	✓	✓		✓	✓				✓	✓		6
Paat & Markham (2019)			✓					✓			✓	3
Pepin & Banyard (2006)			✓	✓		✓		✓				4
Schaefer et al. (2018)				✓				✓				2
Strøm et al. (2013)			✓		✓	✓	✓	✓	✓	✓	✓	8
Tracy et al. (2018)			✓		✓	✓	✓			✓	✓	6
Tyrell & Yates (2017)				✓	✓				✓	½^c^		3.5

*Note.* ✓ = a score of 1; blank cells = a score of 0.

^a^Unclear if criteria met.

^b^Criteria met for some but not all outcomes.

^c^Longitudinal design for most, but not all, participants.
